# Chronic lymphocytic leukemia, a rare cause of spontaneous rupture of the spleen

**DOI:** 10.1016/j.ijscr.2022.107315

**Published:** 2022-06-16

**Authors:** Madani Ayoub, Mohamed Yassine Mabrouk, Hajar Abdelouahab, Imane Kamaoui, Miry Achraf, Siham Hamaz, Khalid Serraj, Jabi Rachid, Bouziane Mohamed

**Affiliations:** a Department of General Surgery, Mohammed VI University Hospital, Faculty of Medicine and Pharmacy, Laboratory of Anatomy, Microsurgery and Surgery, Experimental and Medical Simulation (LAMCESM), Mohammed Ist University, Oujda, Morocco; b Department of Radiology, Mohammed VI University Hospital, Oujda, Morocco; c Department of Anatomopathology, Mohammed VI University Hospital, Oujda, Morocco; d Infectious Diseases, Immunohematology and Cellular Therapy Laboratory, Faculty of Medicine and Pharmacy of Oujda, Mohammed First University of Oujda, Morocco; e Internal Medicine, Immunohematology and Cellular Therapy Laboratory, Faculty of Medicine and Pharmacy of Oujda, Mohammed First University of Oujda, Morocco

**Keywords:** Spontaneous rupture of the spleen, Chronic lymphocytic leukemia, Surgical treatment, Prognosis

## Abstract

**Introduction:**

Spleen Spontaneous Rupture SRS is a rare phenomenon in which the spleen ruptures without associated trauma. This pathology is rarely caused by Chronic Lymphocytic Leukemia.

**Presentation of the case:**

We present a case of a 59-year-old male patient with chronic Lymphocytic Leukemia, who was admitted with an acute abdomen whose clinical and paraclinical examinations revealed a spleen spontaneous rupture. The treatment consisted of a splenectomy.

**Discussion:**

Spontaneous spleen rupture was first described by Rokitansky in 1861 and mentioned in many cases since, the common causes of non-traumatic Splenic rupture include myeloproliferative diseases, vasculitis, and infections. However, Chronic Lymphocytic Leukemia (CLL) remains an obscure cause of splenic rupture that requires unique attention. The diagnosis of splenic rupture should be considered in all patients with hematologic malignancies presenting with abrupt onset of abdominal pain, hemodynamic instability, or acute anemia. The choice between conservative treatment and splenectomy depends on different variables: the etiology of the SRS, the hemodynamic stability, the amount of packed red blood cells transfused. Thus, an interventional approach can be advocated for a spontaneous splenic rupture over nonoperative management. Splenic embolization can provide patients with the advantages of both operative splenectomy and conservative management. The mortality rate from SRS is 12.2 %. Neoplastic pathologies were most significantly associated with fatal outcomes.

**Conclusion:**

The high mortality rate seems to be mainly related to the delayed diagnosis and/or the severity of the underlying pathology. Given its seriousness, it requires a rapid diagnosis and adapted management.

## Highlights

• Spontaneous rupture of the spleen is a rare phenomenon where the spleen ruptures without associated trauma.
• chronic lymphocytic leukemia (CLL) remains an obscure cause of splenic rupture that requires unique attention
• Clinical presentation is usually in the form of a severe abdominal syndrom with hemodynamic instability.
• The choice of treatment: conservative, surgery or embolization depends on several factors.
• The high mortality rate is related to delayed diagnosis and the severity of the underlying pathology.

## 1 Introduction

The spontaneous rupture of the spleen is a rare condition in which the spleen ruptures without associated trauma. It is an unusual complication of malignant hematological disorders. According to the literature only few incidences of spontaneous splenic rupture are caused by chronic lymphocytic leukemia [1].

Non-traumatic splenic rupture is due to many causes, including myeloproliferative diseases, vasculitis, and infections (such as Malaria and infectious Mononucleosis). However, chronic lymphocytic leukemia (CLL) remains an obscure cause of splenic rupture that requires special attention [2].

We present here the case of a 59-year-old male patient with a chronic lymphocytic leukemia history, who got admitted with subcapsular hematoma of the spleen, the surgical intervention consisted of splenectomy and abdominal drainage.

In this article, we represent the clinical manifestations, evaluation, management, and prognosis of this type of diseases. This work has been reported in line with the SCARE criteria 2020 [3].

## 2 Observation

We present the case of a 59-year-old male patient, followed in the onco-hematology department for chronic lymphoid leukemia stage C with negative deletion 17p. A year before his admission, he had benefited from 6 cures during FCR chemotherapy protocol, he never showed up again till he was consulted to the emergency department for acute onset diffuse abdominal pain.

The biological parameters had demonstrated a hyperleukocytosis 13,000 predominantly lymphocytes predominance of 60 %. Microcytic hypochromic anemia at 8.3 g/dl, thrombocytopenia at 98,000 platelets/mm3. Liver and kidney tests came normal and CRP at 23.

**Fig. 1:**
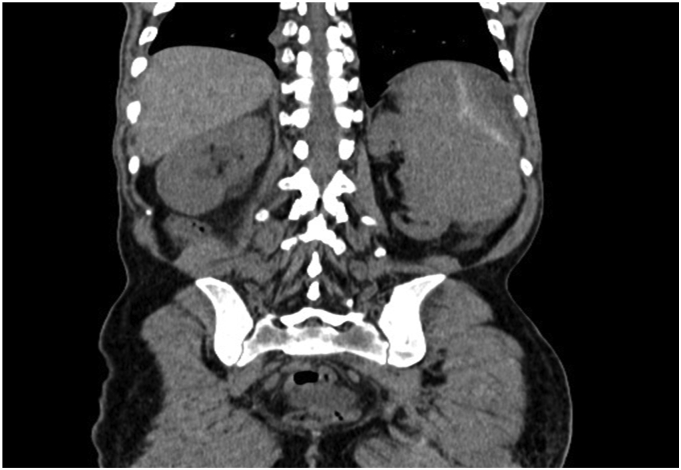
CT image showing splenomegaly with subcapsular hematoma of the spleen.

The abdominal and pelvic CT scan showed Splenomegaly with subcapsular hematoma of the spleen measuring 30 mm in diameter, with an intraperitoneal effusion at the level of the rectouterine pouch, as well as the lumboaortic and coeliomesenteric nodes (Fig. 1).

After multidisciplinary discussion involving visceral surgeons, anesthesiologists, and onco-hematologists, the decision was to proceed with an emergency laparotomy due to a pre-rupture hematoma on a pathological spleen under general anesthesia with endotracheal intubation, the surgical exploration revealed the presence of a giant spleen exceeding the umbilicus below and reaching the midline on the right of multiple intraperitoneal lymphadenopathies and an abdominal effusion of moderate abundance.

**Fig. 2:**
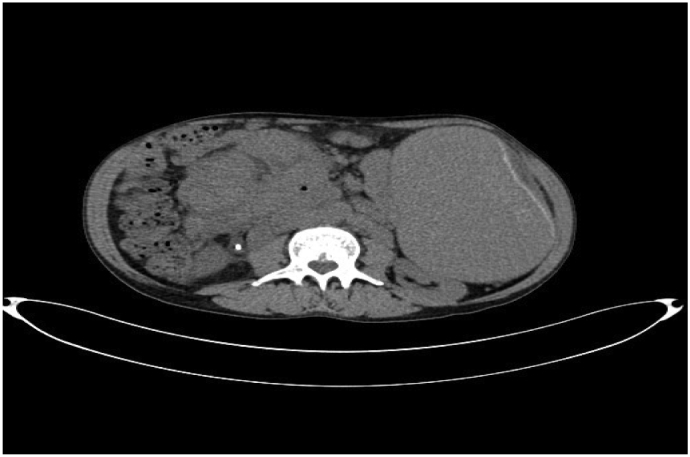
CT image showing splenomegaly with subcapsular hematoma of the spleen.

**Fig. 3:**
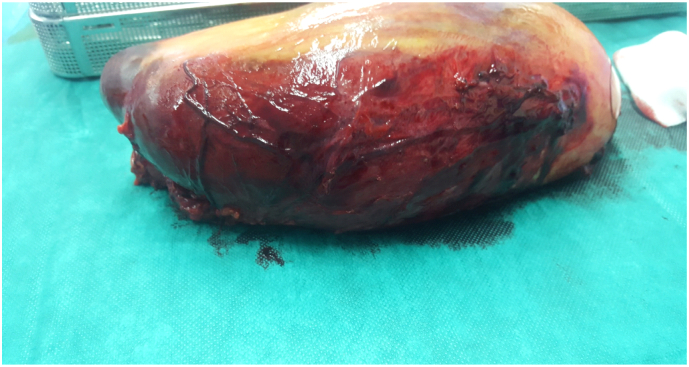
Photograph of the spleen: the parietal side.

**Fig. 4:**
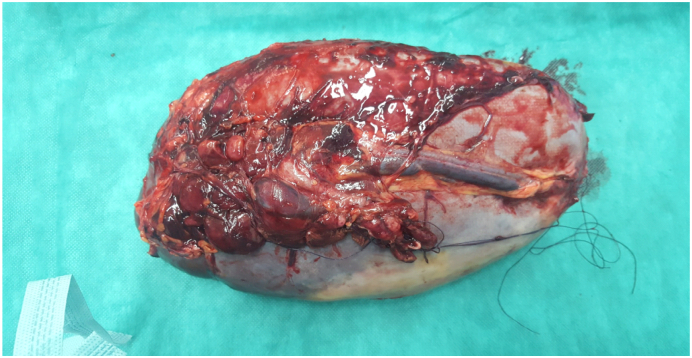
Photograph of the spleen: the visceral side:

**Fig. 5:**
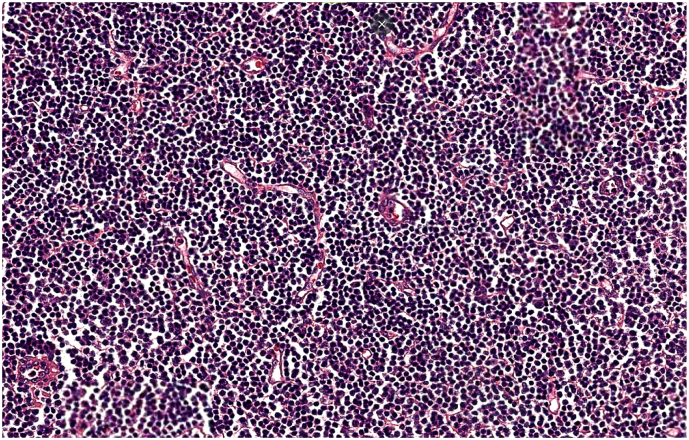
Microphotography showing diffuse effacement of splenic parenchyma by small, mature lymphocytes. They have round nuclei, clumped chromatin with scant cytoplasm. (HE, 200×).

The surgical intervention consisted of splenectomy with abdominal drainage, During the procedure, the patient required a transfusion of 2 units of red blood cells. Postoperatively, the patient had febrile neutropenia complicated by septic shock transferred to intensive care where he received empirical broad-spectrum antibiotic therapy based on 3rd generation cephalosporin in combination with metronidazole and an aminoglycoside then escalation to piperacillin tazobactam following The MASCC clinical score (Multinational Association for Supportive Care in Cancer) and died on the sixth postoperative day. The Macroscopic examination revealed a spleen that weighed 3150 g and is the site of several foci of hemorrhagic infarctions and recent hematomas (Fig. 2, Fig. 3). The histopathologic examination revealed a splenic localization of chronic Lymphoid Leukemia (Fig. 4, Fig. 5).

## 3 Discussion

spontaneous spleen rupture (SRS) was first described by Rokitansky in 1861 and mentioned in many cases since [1]. The term “pathological splenic rupture” was introduced in 1928 by Lange to define the non-traumatic splenic rupture that occurs in a diseased spleen [2].

Renzulli and all summarized 845 patients with atraumatic splenic rupture from previous reports from 1980 to 2008, the analysis revealed that the hematologic cause accounted for 30.3 % of the cases, followed by infection in 27.3 % of the cases, inflammation in 20.0 % of the cases, and drug-related causes (anticoagulants and thrombolytics) in 9.2 % of the cases [4].

In another review by Giagounidis and all analyzing 136 cases of SRS due to hematological diseases, chronic Lymphocytic Leukemia (CLL) was described in 5.8 %, with only 8 cases of SRS [2].

The mechanism of occurrence of spontaneous rupture of the spleen is not specific, Hynes and all. Suggested three mechanisms: mechanical effect of leukemic infiltration in the spleen, especially if the capsule is invaded, splenic infarction with subsequent subcapsular hemorrhage and subsequent rupture of the splenic capsule due to blood coagulation abnormalities. Regarding SRS in chronic lymphocytic leukemia, the incidence is not known, the pathogenesis is still poorly understood, and the mechanisms of leukemic infiltration of coagulopathy and infarction are incriminated [5].

Clinical presentation is usually in the form of a severe abdominal syndrome, which may be accompanied by nonspecific symptoms simulating acute coronary syndrome or acute pancreatitis which delays the diagnosis and the treatment [6]. Patients often present an hypovolemic shock with signs of peritonitis on physical examination [7]. The diagnosis of splenic rupture should be considered in all patients with hematologic malignancies presenting with abrupt onset of abdominal pain, hemodynamic instability or acute anemia.

In most cases, the diagnosis of a splenic rupture or hematoma is made with an improved imaging scan. The results can be obtained quickly which helps diagnose and guide the surgical planning by determining the presence and abondance of intraperitoneal hemorrhage and can sometimes provide information on the etiology of SRS [8].

.The choice of the treatment depends on different variables: the etiology of SRS, hemodynamic stability, the amount of red blood cells transfused, the operability of the patient, the amount of hemoperitoneum as well as the lesion grades according to the World Society for Surgery emergency (WSES) [9].

Examinations of survival associated with spontaneous splenic rupture reported in the literature, 63 % of splenectomies survived [2]. Mortality was much higher with conservative management and was cited at 93 % in a study evaluating 136 cases of pathological splenic rupture reported in the literature [10].

Some investigators use these data to make such aggressive claims that emergency splenectomy is the only possible treatment for these patients [11].

Although successful conservative management has been reported, the mortality rate is still high for this strategy [10]. Although splenectomy can save lives, post-splenectomy infection (OPSI) poses an additional threat to survival in patients treated with chemotherapy, neutropenia and asplenia that may work together to increase the risk of developing severe sepsis [12].

Thus, an interventional approach can be advocated for a spontaneous splenic rupture over nonoperative management. Splenic embolization can provide patients with the advantages of both operative splenectomy and conservative management. Decreasing the blood flow may reduce the risk of delayed splenic rupture and preservation of functional splenic tissue which minimizes the risk of post-splenectomy infection [13].

The study by Renzulli and all calculated the overall mortality rate from SRS to be 12.2 % and it suggests that the major risk factors for death are: splenomegaly and the age over 40 years [4].

The examination of the six major etiologic groups revealed that neoplastic disorders were also significantly associated with a fatal outcome. A traumatic rupture of a normal spleen without etiologic factors (atraumatic-idiopathic splenic rupture). On the other hand, it was associated with a significant decrease in SRS mortality [14].

## 4 Conclusion

The pathological splenic rupture, although rare, must be considered in case of acute abdomen in patients with hemato-oncological pathologies. The high mortality rate seems to be mainly related to delayed diagnosis and/or the severity of the underlying pathology. Given its seriousness, it requires a rapid diagnosis and an adapted management.

## Funding

The author(s) received no financial support for the research, authorship and/or publication of this article.

## Consent

Written informed consent was obtained from the patient for publication of this case report and accompanying images. A copy of the written consent is available for review by the Editor-in-Chief of this journal on request.

## Registration of research studies

1. Name of the registry:
2. Unique identifying number or registration ID:
3. Hyperlink to your specific registration (must be publicly accessible and will be checked):

## Guarantor

Dr. Madani Ayoub

## CRediT authorship contribution statement

▪ Dr. Madani Ayoub: Have written the article, have consulted the patient, prescribed all of tests and prepared the patient for surgery and participated in the surgery

- Dr. Mohamed Yassine Mabrouk: have helped writing the article, data collection.
  ▪ Dr. Hajar Abdelouahab Pr Imane Kamaoui: Interpretation of Radiological data.
- Dr. Miry Achraf: Interpretation of histological data.

Pr Siham Hamaz, Pr Khalid Serraj Sr

▪ : management and follow up of th patient
▪ Pr Jabi Rachid: supervised the writing of manuscript.

- Pr Bouziane Mohamed: (oncology surgery professor): have supervised the writing of the paper, and has been the leader surgeon of the case.

## Declaration of competing interest

No conflit of interest.

## References

[1] Paduri S., Nandu N.S., Brucker T., Roach P., Pant-Purohit M. (2021 Oct 20). Unique case of atraumatic splenic rupture in a patient with chronic lymphocytic leukaemia with Richter's transformation. BMJ Case Rep..

[2] Giagounidis A.A., Burk M., Meckenstock G., Koch A.J., Schneider W. (1996 Dec). Pathologic rupture of the spleen in hematologic malignancies: two additional cases. Ann. Hematol..

[3] Agha R.A., Franchi T., Sohrabi C., Mathew G., Kerwan A., SCARE Group (2020 Dec). The SCARE 2020 guideline: updating consensus Surgical CAse REport (SCARE) guidelines. Int. J. Surg..

[4] Renzulli P., Hostettler A., Schoepfer A.M., Gloor B., Candinas D. (2009 Oct). Systematic review of atraumatic splenic rupture. Br. J. Surg..

[5] Amaki J., Sekiguchi T., Hiraiwa S., Kajiwara H., Kawai H., Ichiki A., Nakamura N., Ando K. (2018 Dec). Three cases of spontaneous splenic rupture in malignant lymphoma. Int. J. Hematol..

[6] Wu M.Y., Kao W.Y., Chan C.Y., Yiang G.T., Liao W.T., Chen C.S. (2019 Oct 18). Spontaneous splenic rupture as a rare initial presentation in an acute lymphoblastic leukemia patient. Diagnostics (Basel).

[7] Zhang Y., Zhang J., Chen T., Zeng H., Zhao B., Zhang Y., Zhou X., Han W., Hu Y., Liu F., Shan Z., Gao W., Zhou H. (2017 Feb). Spontaneous splenic rupture in an acute leukemia patient with splenic tuberculosis: a case report. Mol. Clin. Oncol..

[8] Jain D., Lee B., Rajala M. (2020 May). Atraumatic splenic hemorrhage as a rare complication of pancreatitis: case report and literature review. Clin. Endosc..

[9] Coccolini Federico (2017). Splenic trauma: WSES classification and guidelines for adult and pediatric patients. World J. Emerg. Surg..

[10] Biswas S., Keddington J., McClanathan J. (2006 Nov). Large B-cell lymphoma presenting as acute abdominal pain and spontaneous splenic rupture; a case report and review of relevant literature. World J. Emerg. Surg..

[11] Mobayen M., Yousefi S., Mousavi M. (2020). The presentation of spontaneous splenic rupture in a COVID-19 patient: a case report. BMC Surg..

[12] Leone G., Pizzigallo E. (2015 Oct 13). Bacterial infections following splenectomy for malignant and nonmalignant hematologic diseases. Mediterr. J. Hematol. Infect. Dis..

[13] Jain D., Lee B., Rajala M. (2020 May). Atraumatic splenic hemorrhage as a rare complication of pancreatitis: case report and literature review. Clin. Endosc..

[14] Aubrey-Bassler F.K., Sowers N. (2012 Aug). 613 cases of splenic rupture without risk factors or previously diagnosed disease: a systematic review. BMC Emerg. Med..

